# Hepatic T1 Mapping in Takotsubo Syndrome: A Preliminary Imaging Insight into the Cardiohepatic Axis

**DOI:** 10.3390/life15091335

**Published:** 2025-08-22

**Authors:** Riccardo Cau, Alessandro Pinna, Maria Francesca Marchetti, Jasjit S. Suri, Roberta Montisci, Luca Saba

**Affiliations:** 1 Department of Radiology, Azienda Ospedaliero Universitaria (A.O.U.), di Cagliari–Polo di Monserrato s.s. 554 Monserrato, 09045 Cagliari, Italy; alessandpi3@gmail.com (A.P.); lucasaba@tiscali.it (L.S.); 2 Department of Medical Sciences and Public Health, University of Cagliari, 09045 Cagliari, Italy; mfrancescacardio@gmail.com (M.F.M.); rmontisc@gmail.com (R.M.); 3 Department of Medical Sciences and Public Health, Clinical Cardiology Unit, University of Cagliari, 09045 Cagliari, Italy; 4 Department of ECE, Idaho State University, Pocatello, ID 83209, USA; jsuri@comcast.net; 5 Department of CE, Graphics Era Deemed to be University, Dehradun 248002, India; 6 University Center for Research & Development, Chandigarh University, Mohali 140413, India; 7 Symbiosis Institute of Technology, Nagpur Campus, Symbiosis International (Deemed University), Pune 440008, India; 8 Stroke Diagnostic and Monitoring Division, AtheroPoint^TM^, Roseville, CA 95661, USA

**Keywords:** takotsubo syndrome, cardiovascular magnetic resonance, T1 mapping, liver

## Abstract

**Background:** Takotsubo syndrome (TS) is an acute heart failure characterized by transient systolic dysfunction of the left ventricle (LV). Given the complex cardiohepatic interactions in heart failure, the purpose of this study was to examine the role of hepatic T1 mapping in TS patients as an imaging biomarker of the cardiohepatic axis and to explore its correlation with demographics, laboratory data, and cardiovascular magnetic resonance (CMR) findings. **Methods:** In this retrospective pilot study, CMR was performed in 62 consecutive patients with TS (54 females, 73.47 ± 9.88 years). Additionally, 24 age- and sex-matched control subjects were included (20 females, 69.67 ± 6.88 years). A dedicated CMR software (CV42 6.0, CVI42, Circle Cardiovascular Imaging Inc., Calgary, AB, Canada) was used to assess atrial and ventricular strain parameters, as well as parametric mapping, including hepatic T1 mapping. **Results:** TS patients exhibited significantly higher hepatic T1 mapping values compared with the age-, sex-, and cardiovascular risk factor-matched control group (499.80 ± 141.86 vs. 425.26 ± 51.91, *p* = 0.017). In multivariable analysis, hepatic T1 mapping was independently associated with right ventricular (RV) longitudinal strain (β coefficient = 2.936, *p* = 0.007) and N-terminal pro-B-type natriuretic peptide (β coefficient = 2.395, *p* = 0.024). **Conclusions:** In this pilot study, hepatic T1 mapping was elevated in TS patients, suggesting its potential role as an imaging biomarker of cardiohepatic interaction. Hepatic T1 also showed independent associations with RV longitudinal strain and N-terminal pro-B-type natriuretic peptide, both well-known markers of adverse outcomes in TS. These preliminary findings warrant validation in larger studies.

## 1. Introduction

Takotsubo syndrome (TS) is a reversible acute heart failure characterized by transient left ventricle (LV) systolic impairment [1,2,3,4,5]. Although the incidence of TS is increasing and both its short-term and long-term complications are becoming better understood, its pathogenesis remains incompletely understood. Emerging evidence suggests that TS may involve a multi-organ component, extending beyond the myocardium [6,7,8].

The heart and liver are tightly interconnected: cardiac dysfunction can lead to hepatic impairment through venous congestion and reduced perfusion, while hepatic dysfunction may worsen systemic and cardiac outcomes [9]. Previous research has demonstrated that impairment of the cardiohepatic axis is an independent predictor of cardiovascular events and mortality across different cardiovascular diseases [10,11,12,13,14].

Cardiovascular magnetic resonance (CMR) is a pivotal non-invasive imaging modality for the comprehensive assessment of cardiac function and myocardial tissue abnormalities in TS, providing both diagnostic and prognostic insights [4,15,16,17,18,19,20,21,22,23,24,25]. Recently, T1 relaxation time has proven useful for characterizing the myocardium and assessing liver tissue properties in various cardiovascular diseases, offering insights into passive liver congestion during acute cardiac dysfunction [14,26,27,28]. Indeed, the close anatomical proximity of the liver to the heart inherently allows CMR imaging to capture a significant portion of the liver during routine parametric mapping sequences, without requiring additional sequences.

During the acute phase of TS, acute heart failure can lead to reduced tissue perfusion and increased venous pressure, resulting in venous congestion that impairs hepatic blood flow and potentially causes varying degrees of liver involvement [9]. Detecting early liver involvement in TS could therefore provide a window into the severity of acute heart failure, identify patients at higher risk for complications, and inform tailored therapeutic strategies. However, to the best of our knowledge, no previous studies have evaluated the potential cardiohepatic interaction through T1 mapping in TS.

Therefore, this study aimed to investigate the role of hepatic T1 mapping in TS patients as an imaging biomarker of the cardiohepatic axis and to explore its correlation with demographics, laboratory data, and CMR findings.

## 2. Materials and Methods

### 2.1. Study Population

From 3 March 2017 to 31 December 2023, 62 patients with TS who met the criteria outlined in the Position Statement of the European Society of Cardiology Heart Failure Association were included in the study [1].

The diagnostic criteria for TS included regional wall motion abnormalities extending beyond a single epicardial vascular territory, typically preceded by an identifiable stressful trigger, in the absence of culprit atherosclerotic disease on invasive coronary angiography. Additional supportive findings were new ECG abnormalities, elevated natriuretic peptide levels with only modest troponin elevation, and recovery of LV function at follow-up.

Exclusion criteria were: age < 18 years, previous myocardial infarction, pre-existing cardiomyopathy, suspected or confirmed irreversible myocardial damage, and known hepatic or biliary disease.

In addition, 24 age- and sex-matched control subjects underwent CMR for evaluation of possible scar-related ventricular tachycardia. Controls were included only if CMR showed no structural abnormalities and if they had no known history of cardiovascular, hepatic, or biliary disease.

Cardiovascular risk factors were obtained from medical records. Hypertension was defined as systolic blood pressure ≥ 140 mmHg or diastolic blood pressure ≥ 90 mmHg at rest on more than two separate occasions, or the use of antihypertensive medication [29]. Smoking status was classified as current smokers or never smokers. Serum cholesterol levels were measured according to the standard in-house laboratory protocol. Diabetes mellitus was defined according to World Health Organization criteria [30] or based on a prior diagnosis of type 2 diabetes. Obesity was defined as a body mass index > 30, in line with the World Health Organization criteria [31].

Approval for this retrospective cross-sectional study was obtained from the Institutional Review Board, and the requirement for patient consent was waived due to the retrospective design.

**Figure 1:**
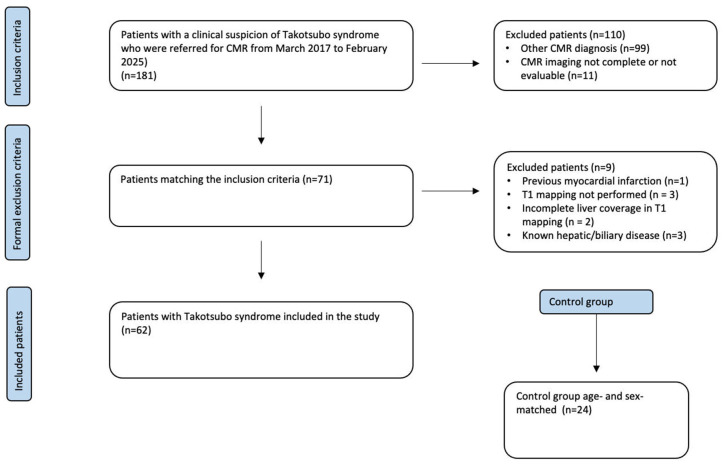
Flowchart of the patients enrolled.

A flowchart illustrating the application of inclusion and exclusion criteria is provided in Figure 1.

### 2.2. CMR Acquisition

CMR scans were acquired at 4.3 ± 2.7 days (median = 4 days, range = 1–9 days) after symptom onset using a Philips Achieva dStream 1.5 T scanner system (Philips Healthcare, Best, The Netherlands). The CMR protocol used for both the TS and control groups included short- and long-axis cine-CMR sequences, short-axis T1 and T2 mapping (obtained on three short-axis slices at the base, mid-ventricular, and apex), and short- and long-axis late gadolinium enhancement (LGE) imaging. All cine-images were acquired using a balanced steady-state free precession with retrospective gating during expiratory breath-hold maneuvers (TE: 1.7 mssec; TR: 3.4 msec/flip-angle: 45°, section thickness  =  8 mm). Imaging was performed in both long-axis views (two-, three-, and four-chamber) and short-axis views with full ventricular coverage from base to apex. A stack of 10–15 contiguous short-axis slices was obtained to cover the entire left and right ventricles (LV and RV, respectively).

T2 mapping was performed before the administration of contrast media on three representative short-axis slices (at the base, mid-ventricular, and apex, respectively) using a single-breath-hold, black-blood prepared ECG-triggered, spin-echo multiecho sequence.

The perfusion sequences were performed after contrast media injection (Gadovist, Bayer Healthcare, Berlin, Germany) with a dose of 0.15 mL per kg on three representative short-axis slices (at the base, mid-ventricular, and apex, respectively) body weight using an inversion recovery prepared echo-planar sequence (prepulse delay: 100 ms; TE: 1.1 mssec; TR: 1.8 msec/flip-angle: 30°, FOV, 300 × 300 mm^2^). Each set of first-pass perfusion images was acquired in 80 cardiac cycles.

LGE imaging was performed 10–12 min after contrast media injection (Gadovist, Bayer Healthcare; 0.15 mL/kg body weight) using phase-sensitive inversion recovery (PSIR) sequences acquired in both short- and long-axis orientations. The appropriate inversion time was determined individually for each patient using the Look-Locker technique.

### 2.3. CMR Image Post-Processing

**Figure 2:**
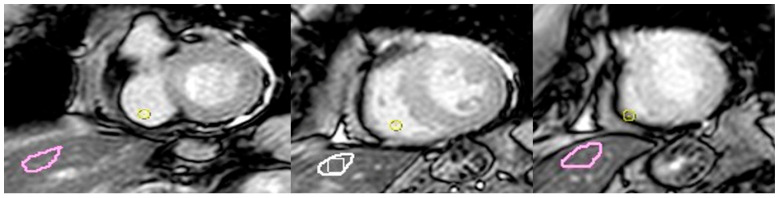
An example of hepatic T1 mapping quantification in a patient with TS. Hepatic T1 mapping was performed by averaging values from three regions of interest (purple circle) placed within the liver parenchyma (each approximately 2 cm^2^). Fat and blood vessels were meticulously excluded by cross-referencing T1 mapping images with corresponding steady-state free precession cine images to ensure accurate tissue sampling.

We used the commercially available software Circle CVI42 (CV42 6.0, CVI42, Circle Cardiovascular Imaging Inc., Calgary, AB, Canada) for myocardial strain analysis as well as T1 and T2 mapping. All data were anonymized and handled in accordance with privacy regulations to ensure the protection of patient confidentiality. Hepatic T1 mapping was conducted by an observer with 10 years of experience in cardiovascular imaging, who averaged values from three regions of interest (ROIs) drawn within the liver parenchyma, each approximately 2 cm^2^ in size, as previously described [14]. Fat and blood vessels were carefully excluded from the ROIs by cross-referencing the T1 mapping images with the corresponding steady-state free precession cine images (Figure 2).

LV volumes and function were determined from short-axis cine images using an automatic computation to outline the endocardial and epicardial borders throughout the cardiac cycle. The accuracy of tracking and contouring was visually inspected, manually adjusted when necessary, and applied consistently across all patients. LV end-diastolic volume and end-systolic volume were derived, and LV ejection fraction (EF) was calculated as: (LV end-diastolic volume − LV end-systolic volume)/LV end-diastolic volume × 100%.

Offline CMR feature tracking analyses were performed to assess global longitudinal strain (GLS), global radial strain (GRS), and global circumferential strain (GCS) using a 16-segment, software-generated 2D model. Longitudinal strain was derived from two-, three-, and four-chamber long-axis views, while radial and circumferential strain was obtained from short-axis views in all patients. The epi- and endocardial borders were traced in end-diastole on all images, after which an automatic computation was initiated to outline the borders throughout the cardiac cycle. The quality of tracking and contouring was visually validated and manually corrected as needed.

Atrial deformation analyses were also performed offline. The left atrial (LA) endocardial borders were manually traced at minimum atrial volume on two-, three-, and four-chamber long-axis cine views, excluding the LA appendage and pulmonary veins. After manual segmentation, the software automatically tracked the myocardial borders throughout the entire cardiac cycle. The quality of tracking and contouring was visually validated and manually corrected by a radiologist with three years of experience in cardiac imaging. The strain curve included three peaks: reservoir, conduit, and booster strain.

The extent of LGE was evaluated both qualitatively and quantitatively using phase-sensitive images. Briefly, myocardial segments were identified and assessed. For quantitative analysis, the epicardial and endocardial borders were manually traced on each short-axis slice. A region of interest (ROI) was manually placed in the visually normal myocardium without LGE. Myocardial regions were defined as LGE-positive if the signal intensity exceeded 5 standard deviations above the mean signal of the reference ROI [32,33]. The extent of LGE was expressed as a percentage of the LV mass.

### 2.4. Statistical Analysis

Continuous variables are presented as mean ± standard deviation. Comparisons of continuous data were performed using the independent samples *t*-test or Mann–Whitney U test; Kolmogorov–Smirnov tests were used to check continuous variables for normal distribution. Categorical variables were compared by using the chi-square test or Fisher’s exact test, as appropriate.

The association between the hepatic T1 mapping, clinical parameters, and TS was analyzed using multivariable linear regression. A *p*-value < 0.05 was considered statistically significant. All statistical analysis was performed using IBM SPSS Statistics version 22 (SPSS Inc., Chicago, IL, USA).

## 3. Results

### 3.1. Baseline Characteristics, Clinical, and CMR Parameters in TS

A total of 62 patients with TS (mean age 73.47 ± 9.88 years, 87% female) and 24 control subjects (mean age 69.67 ± 6.88 years, 83% female) were included. Among the patients with TS, 58 (93%) exhibited the classic apical ballooning pattern, while three (4%) presented with a mid-ventricular variant and one (2%) with a reverse Takotsubo pattern.

No significant differences were found in age, sex, or cardiovascular risk factors among the enrolled patients. The baseline characteristics of the enrolled patients are shown in Table 1.

TS patients demonstrated a lower LV ejection fraction (49.49% ± 11.01 vs. 61.28% ± 6.42, *p* = 0.001). No other differences were observed in terms of LV and right ventricular (RV) volumes and function. Higher T2 mapping values (66.96 ms ± 5.83 vs. 53.18 ± 3.62, *p* = 0.001) were observed in TS patients compared to control subjects.

Regarding myocardial strain parameters, TS patients exhibited impaired LV and RV longitudinal strain (−11.41 ± 4.33 vs. −15.5 ± 2.52, *p* = 0.001; −16.67 ± 6.33 vs. −19.97 ± 4.99, *p* = 0.026, respectively), LV circumferential strain (−14.07 ± 6.11 vs. −16.85 ± 2.99, *p* = 0.001), and left and right atrial reservoir and conduit strain (23.03 ± 8.69 vs. 32.24 ± 8.99, *p* = 0.001; 11.43 ± 6.05 vs. 18.87 ± 6.46, *p* = 0.001; 28.76 ± 14.18 vs. 36.67 ± 11.90, *p* = 0.020; 17.20 ± 10.23 vs. 21.06 ± 8.16, *p* = 0.047, respectively). Among patients with TS, CMR detected LGE in 19 cases (30%) using the 5-SD threshold, with enhancement patterns involving the full thickness of myocardial segments corresponding to wall motion abnormalities. No LGE was observed in healthy controls. Table 2.

### 3.2. Hepatic T1 Mapping and Its Determinants in TS Patients

**Figure 3:**
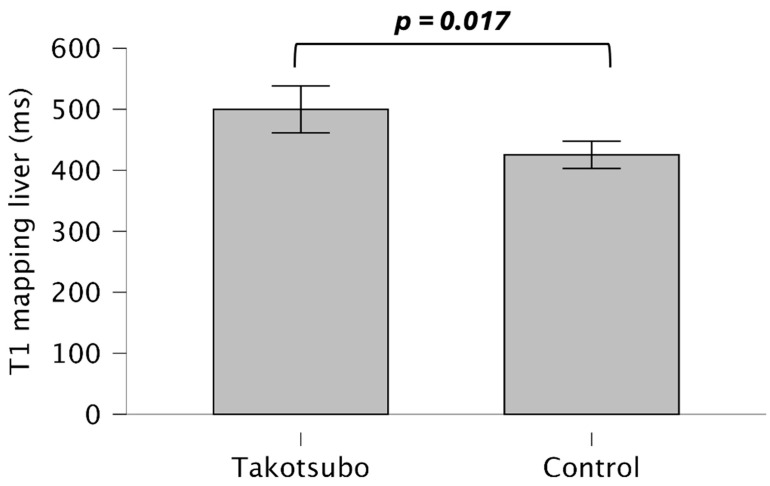
Hepatic T1 mapping in Takotsubo and healthy controls.

TS patients demonstrated higher T1 liver mapping value (499.80 ± 141.86 vs. 425.26 ± 51.91, *p* = 0.017) in comparison with control subjects (Figure 3).

Univariable analysis revealed that obesity (β = −2.591, *p* = 0.013), NT-proBNP (β = 2.578, *p* = 0.015), LV end-diastolic indexed volume (β = 2.551, *p* = 0.014), LV end-systolic indexed volume (β = 2.984, *p* = 0.004), RV end-diastolic indexed volume (β = 3.525, *p* = 0.001), RV end-systolic indexed volume (β = 3.446, *p* = 0.001), RV stroke volume indexed (β = 3.182, *p* = 0.002), RV global longitudinal strain (β = 4.414, *p* = 0.001), and right atrial conduit function (β = 1.992, *p* = 0.04) were associated with T1 liver mapping.

On multivariable analysis, after adjusting for all significant variables in the univariate analysis, NT-proBNP (β coefficient = 2.395, *p* = 0.024) and RV longitudinal strain (β coefficient = 2.936, *p* = 0.007) remained independently associated with T1 liver mapping (Table 3).

## 4. Discussion

The main findings of this pilot study were as follows: (1) hepatic T1 mapping values were higher in TS patients compared to the control group; (2) an increase in hepatic T1 mapping values was independently associated with RV GLS; (3) an increase in hepatic T1 mapping values was independently positively correlated with NT-BNP. Our findings support the hypothesis of multi-organ involvement during the acute phase of Takotsubo syndrome, with passive liver congestion resulting from cardiohepatic interaction, and highlight its relationship with well-established markers of adverse cardiovascular events in TS, including NT-BNP and right ventricular involvement.

Previous studies have investigated the potential of liver T1 mapping as a tool for evaluating tissue properties beyond the myocardium, highlighting its role in assessing hepatic involvement in cardiovascular disease. Supporting the clinical relevance of liver T1 imaging, data from the Multi-Ethnic Study of Atherosclerosis (MESA) demonstrated that individuals with a history of atrial fibrillation, heart failure, or coronary artery disease exhibited significantly prolonged native hepatic T1 relaxation times compared with disease-free participants [34]. Consistently, similar associations have been reported in patients with dilated and ischemic cardiomyopathy, in whom elevated liver T1 values correlated with the presence and severity of heart failure [14,35].

To the best of our knowledge, this is the first study to explore liver relaxometry as a marker of the cardiohepatic interaction in TS patients.

During the acute phase of Takotsubo syndrome, transient systolic dysfunction of the left ventricle can lead to acute heart failure, triggering a cascade of effects throughout the body. A key consequence is reduced tissue perfusion, combined with increased venous pressure, which results in venous congestion that impairs hepatic blood flow. This lack of proper perfusion can lead to a range of liver issues, from mild biochemical abnormalities to more severe dysfunction. Non-invasive assessment of splanchnic organ hypoperfusion is crucial for evaluating the hemodynamic status and tailoring medical therapy. CMR has gained increasing importance for structural and functional assessment during the course of TS. Furthermore, because of the liver’s close anatomical proximity to the heart, CMR offers the advantage of simultaneously assessing both cardiac and hepatic tissues without the need for additional imaging sequences or acquisition time [14,26,27,28]. During acute heart failure, hepatic congestion from impaired cardiac function can significantly alter the liver’s proton environment with consequent changes in T1 mapping values [9,36].

Notably, TS patients exhibit higher hepatic T1 mapping values compared to controls, suggesting potential liver involvement during the course of TS. This aligns with prior studies emphasizing a bidirectional relationship between cardiac dysfunction and hepatic injury [14], potentially mediated by systemic hemodynamic alterations and neurohormonal dysregulation.

Elevated T1 liver values in TS patients may reflect subclinical hepatic congestion to the acute cardiac dysfunction typical of TS [9].

Interestingly, NT-proBNP levels and RV longitudinal strain were independently associated with hepatic T1 mapping values in the multivariable analysis.

Natriuretic peptides, released by cardiomyocytes in response to cardiac volume or pressure overload, play an important role in the diagnosis and prognosis of TS patients [1] and are significantly increased in those with liver dysfunction [37]. Therefore, their correlation with hepatic T1 mapping further underscores the interconnected relationship between cardiac and hepatic function in TS.

Our findings demonstrated an independent association between RV involvement and higher hepatic T1 mapping values, which are linked to the complex cardiohepatic interactions that occur during heart failure. Although the exact etiopathology of TS is not fully understood, one may speculate that RV dysfunction in TS leads to increased central venous pressure, resulting in venous congestion within the systemic circulation [9]. The liver, as one of the first affected splanchnic organs in congestive heart failure, is particularly susceptible to this impaired venous return [9].

RV involvement in TS patients has been associated with a more severe disease course and worse clinical presentation [15,38,39]. Therefore, the assessment of parameters linked to right ventricular dysfunction is useful in clinical practice.

The integration of hepatic T1 mapping as an imaging biomarker to non-invasively capture cardiohepatic interactions into routine CMR protocols may have different clinical implications for the management of TS. First, it provides a non-invasive tool to detect subclinical hepatic involvement, which may reflect systemic congestion and multi-organ stress during the acute phase. Second, hepatic T1 values may serve as an adjunctive biomarker for risk stratification. Their independent association with right ventricular longitudinal strain and NT-proBNP, both well-established indicators of adverse prognosis, underscores the potential utility of hepatic T1 in identifying patients at higher risk of adverse events, thereby guiding closer monitoring, more aggressive supportive interventions, or early referral to specialized care.

These findings support the concept that TS is not solely a cardiac condition but may involve systemic impairment, including subclinical hepatic congestion. Identifying such extracardiac involvement could be crucial for refining clinical management. Importantly, recognizing early signs of systemic dysfunction—particularly hepatic involvement—may enable clinicians to stratify patients more effectively and implement a tailored therapeutic approach. This could include not only optimized hemodynamic and cardiac support but also targeted interventions to alleviate hepatic congestion or dysfunction (e.g., N-acetylcysteine [40,41]), thereby promoting multi-organ recovery through a multidisciplinary strategy.

Additionally, because hepatic T1 mapping is derived from parametric sequences routinely acquired for myocardial tissue characterization, it adds clinical value without requiring additional scan time or contrast administration. Future longitudinal studies are warranted to assess whether hepatic T1 mapping can predict adverse outcomes, monitor therapeutic response, or guide integrated cardiohepatic management strategies in TS. Ultimately, incorporating hepatic imaging biomarkers may represent a meaningful step toward a more comprehensive and personalized approach to the care of patients with TS.

This study has several limitations that should be acknowledged. First, the relatively small sample size represents a significant limitation of our study, as it may not only restrict the generalizability of the findings but also reduce the robustness and statistical power of our analyses. While our results provide novel and promising insights, they should be regarded as preliminary. Validation in larger, preferably multicenter cohorts will be essential to confirm these observations.

Second, the study employed a cross-sectional design, which precludes the assessment of the predictive value of hepatic T1 for adverse cardiovascular events in patients with TS. Additionally, changes in hepatic T1 values over time were not examined. Future longitudinal studies are required to evaluate the prospective association of this CMR parameter with patient outcomes and to investigate quantitative changes over time. It should also be acknowledged that hepatic T1 can be influenced by several other factors, including fibrosis, inflammation, and iron overload. Because multiparametric liver assessment was not performed, the specificity of our findings for passive congestion cannot be definitively established. Future studies integrating multiparametric liver imaging and biochemical profiling will be essential to disentangle these mechanisms and to better characterize the nature and specificity of hepatic T1 alterations in TS.

Third, a comparison with patients affected by other forms of acute or chronic heart failure was not included, which limits the specificity of our findings. Future studies involving well-defined comparator groups are needed to better delineate the diagnostic and pathophysiological specificity of hepatic T1 changes in this context.

Lastly, although individuals with known hepatic or biliary disease were excluded, we did not perform direct fat quantification or assess subclinical liver involvement, which may have influenced hepatic T1 values. In addition, CMR scans were acquired with considerable variability after symptom onset, which could have affected hepatic T1 and other imaging parameters, as the degree of liver congestion and myocardial changes can evolve rapidly during the acute phase of TS. Further investigations incorporating multiparametric liver imaging, biochemical profiling, and standardized imaging timing are warranted to address these potential confounding factors.

## 5. Conclusions

In this pilot study, hepatic T1 mapping, acquired during a standard CMR protocol, may provide preliminary information about the concomitant presence of liver congestion in TS. Further clinical studies, including patients with other forms of acute or chronic heart failure, are warranted to better elucidate the interplay between cardiac and hepatic dysfunction in TS.

## Figures and Tables

**Table 1:** Baseline characteristics of patients with TS and normal controls.

Variables	Takotsubo	Control	*p*-Value
Sex (female), n (%)	54 (87%)	20 (83%)	0.656
Age (years)	73.47 ± 9.88	69.67 ± 6.88	0.129
Hypertension, n (%)	33 (53%)	15 (62%)	0.063
Dyslipidemia, n (%)	18 (27%)	7 (29%)	0.060
Smoke, n (%)	9 (14%)	4 (17%)	0.634
Obesity, n (%)	6 (9%)	2 (8%)	0.800
Diabetes, n (%)	7 (11%)	4 (16%)	0.584
Family History of CAD, n (%)	10 (16%)	3 (12%)	0.122
**Triggers**			
Emotional Trigger, n (%)	33 (53%)	/	/
Physical Trigger, n (%)	22 (35%)	/	/
**Lab data**			
Troponine	3643.36 ± 2275.5	/	/
NT-proBNP	6631.55 ± 7025	/	/

Abbreviations: CAD, coronary artery disease; NT-proBNP, N-terminal-pro hormone brain natriuretic peptides.

**Table 2:** CMR findings of patients with TS and normal controls.

Variables	Takotsubo	Control	*p*-Value
LVEF, %	49.49 ± 11.01	61.28 ± 6.42	**0.001**
LVEDV/BSA, mL/m^2^	84.54 ± 40.17	84.63 ± 20	0.322
LVESV/BSA, mL/m^2^	40.58 ± 24.60	33.63 ± 11.24	0.206
LVSV/BSA, mL/m^2^	43.80 ± 19.37	50.99 ± 10.78	0.103
RVEF, %	58.23 ± 8.09	58.60 ± 7.09	0.860
RVEDV/BSA, mL/m^2^	63.54 ± 25.43	73.67 ± 22.99	0.151
RVESV/BSA, mL/m^2^	26.43 ± 11.45	33.45 ± 14.18	0.226
RVSV/BSA, mL/m^2^	37.12 ± 15.67	41.16 ± 10.49	0.058
GLS, %	−11.41 ± 4.33	−15.5 ± 2.52	**0.001**
GCS, %	−14.07 ± 6.11	−16.85 ± 2.99	**0.027**
GRS, %	24,21 ± 9.84	28.10 ± 7.53	0.096
RV GLS, %	−16.67 ± 6.33	−19.97 ± 4.99	**0.026**
Reservoir, %	23.03 ± 8.69	32.24 ± 8.99	**0.001**
Conduit, %	11.43 ± 6.05	18.87 ± 6.46	**0.001**
Booster, %	11.74 ± 8.19	13.36 ± 4.82	0.599
RA Reservoir, %	28.76 ± 14.18	36.67 ± 11.90	**0.020**
RA Conduit, %	17.20 ± 10.23	21.06 ± 8.16	**0.047**
RA Booster, %	10.96 ± 9.99	15.54 ± 7.40	0.051
T2 STIR, number of segments (n)	7.45 ± 2.80	/	/
LGE present, n (%)	19 (30%)	/	/
T2 mapping, ms	66.96 ± 5.83	53.18 ± 3.62	**0.001**
T1 liver	499.80 ± 141.86	425.26 ± 51.91	**0.017**

Abbreviations: BSA, body surface area; EDV, end-diastolic volume; ESV, end-systolic volume; GCS, global circumferential strain; GLS, Global longitudinal strain; GRS, Global radial strain; LGE, late gadolinium enhancement; LV, left ventricle; LVEF, left ventricle ejection fraction; RA, right atrium; RV, right ventricle; RVEF, right ventricle ejection fraction; STIR, short tau inversion recovery; SV, stroke volume.

**Table 3:** Univariable and multivariable determinants of hepatic T1 mapping in patients with Takotsubo syndrome.

Variables	Univariable		Multivariable	
	β Coefficient	*p* Values	β Coefficient	*p* Values
Sex	1.332	0.189		
Age	1.006	0.319		
Hypertension	−0.252	0.802		
Dyslipidemia	1.275	0.208		
Smoke	−1.240	0.221		
Obesity	−2.591	**0.013**	−1.914	0.064
Diabetes	−0.383	0.703		
Family History of CAD	1.553	0.127		
Emotional Trigger	−0.255	0.823		
Physical Trigger	0.428	0.672		
Troponine	−0.554	0.584		
NT-proBNP	2.578	**0.015**	2.395	**0.024**
LVEF, %	0.621	0.537		
LVEDV/BSA, mL/m^2^	2.551	**0.014**	0.004	0.997
LVESV/BSA, mL/m^2^	1.861	0.068		
LVSV/BSA, mL/m^2^	2.984	**0.004**	0.446	0.660
RVEF, %	−0.760	0.450		
RVEDV/BSA, mL/m^2^	3.525	**0.001**	0.387	0.702
RVESV/BSA, mL/m^2^	3.446	**0.001**	0.403	0.676
RVSV/BSA, mL/m^2^	3.182	**0.002**	0.375	0.711
GLS, %	−1.221	0.228		
GCS, %	−0.498	0.620		
GRS, %	0.554	0.582		
RV GLS, %	4.314	**0.001**	2.936	**0.007**
Reservoir, %	−0.247	0.806		
Conduit, %	1.235	0.222		
Booster, %	−1.040	0.303		
RA Reservoir, %	1.410	0.164		
RA Conduit, %	1.992	**0.04**	1.794	0.085
RA Booster, %	−0.288	0.775		
T2 STIR segments	0.572	0.570		
LGE	−0.315	0.730		
T2 mapping, ms	−0.284	0.807		

Multivariable analysis was adjusted for factors that were statistically significant in the univariable analysis. BSA, body surface area; CAD, coronary artery disease; EDV, end-diastolic volume; ESV, end-systolic volume; LGE, late gadolinium enhancement; LV, left ventricle; LVEF, left ventricle ejection fraction; GCS, global circumferential strain; GLS, global longitudinal strain; GRS, global radial strain; NT-proBNP, N-terminal-pro hormone brain natriuretic peptides; RA, right atrium; RV, right ventricle; RVEF, right ventricle ejection fraction; STIR, short tau inversion recovery; SV, stroke volume.

## Data Availability

The data will be made available upon reasonable request to the corresponding author.
